# The Electrophysiology of Presynaptic Congenital Myasthenic Syndromes With and Without Facilitation: From Electrodiagnostic Findings to Molecular Mechanisms

**DOI:** 10.3389/fneur.2019.00257

**Published:** 2019-03-19

**Authors:** Stefan Nicolau, Margherita Milone

**Affiliations:** Department of Neurology, Mayo Clinic, Rochester, MN, United States

**Keywords:** congenital myasthenic syndromes, electromyography, facilitation, neuromuscular junction, repetitive nerve stimulation

## Abstract

Congenital myasthenic syndromes (CMS) are a group of inherited disorders of neuromuscular transmission most commonly presenting with early onset fatigable weakness, ptosis, and ophthalmoparesis. CMS are classified according to the localization of the causative molecular defect. CMS with presynaptic dysfunction can be caused by mutations in several different genes, including those involved in acetylcholine synthesis, its packaging into synaptic vesicles, vesicle docking, and release from the presynaptic nerve terminal and neuromuscular junction development and maintenance. Electrodiagnostic testing is key in distinguishing CMS from other neuromuscular disorders with similar clinical features as well as for revealing features pointing to a specific molecular diagnosis. A decremental response on low-frequency repetitive nerve stimulation (RNS) is present in most presynaptic CMS. In CMS with deficits in acetylcholine resynthesis however, a decrement may only appear after conditioning with exercise or high-frequency RNS and characteristically displays a slow recovery. Facilitation occurs in CMS caused by mutations in *VAMP1, UNC13A, SYT2, AGRN, LAMA5*. By contrast, facilitation is absent in the other presynaptic CMS described to date. An understanding of the underlying molecular mechanisms therefore assists the interpretation of electrodiagnostic findings in patients with suspected CMS.

## Introduction

**Figure 1:**
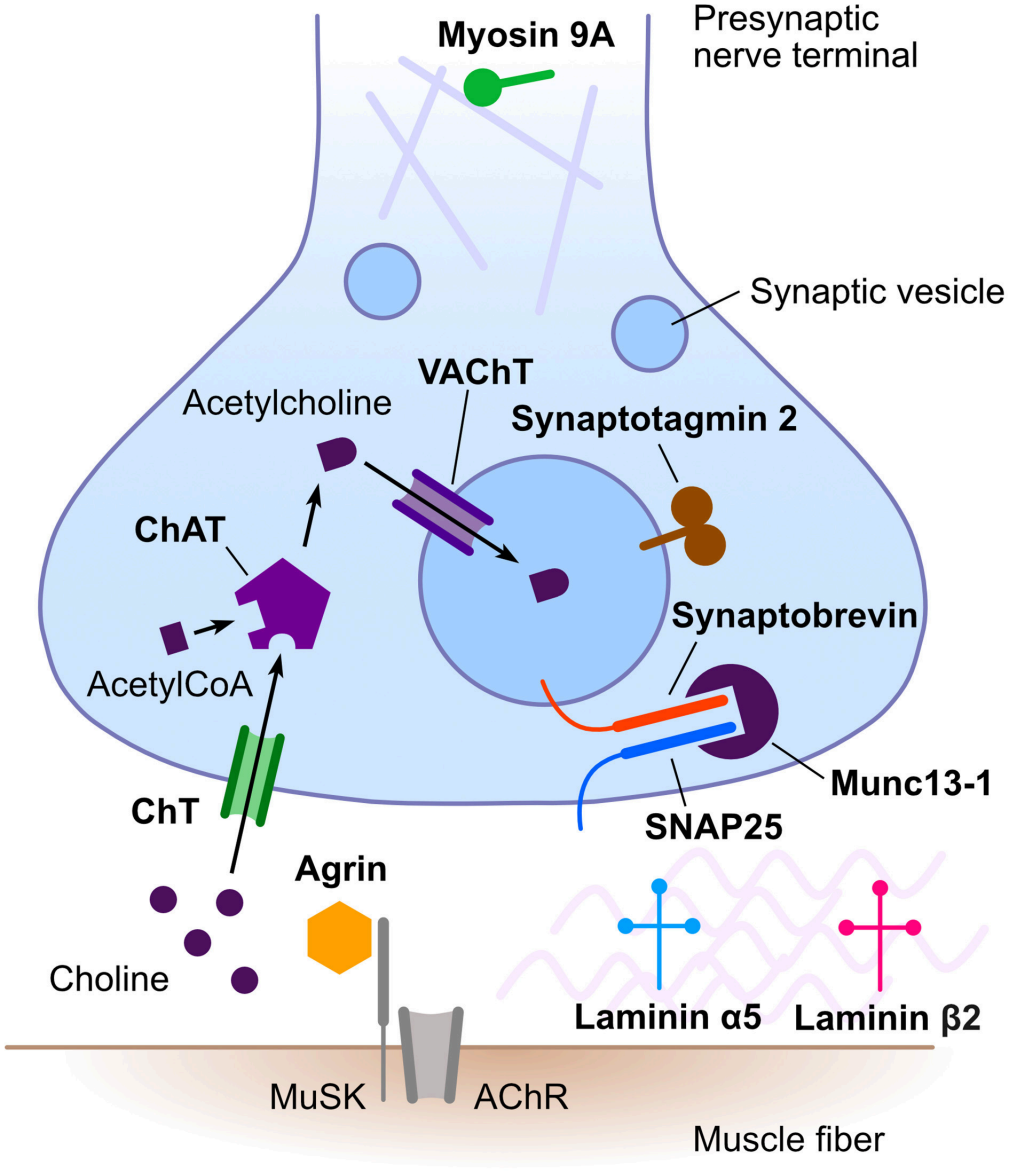
Schematic representation of the neuromuscular junction emphasizing proteins involved in presynaptic congenital myasthenic syndromes. Names in bold designate proteins implicated in presynaptic congenital myasthenic syndromes. AChR, acetylcholine receptor; ChAT, choline acetyltransferase; ChT, high-affinity presynaptic choline transporter; MuSK, muscle-specific kinase; SNAP25, synaptosomal-associated protein 25**;** VAChT, vesicular acetylcholine transporter. Rabphilin 3A is not illustrated, as its precise role in neuromuscular transmission remains to be established.

Congenital myasthenic syndromes (CMS) are a heterogeneous group of rare inherited disorders of neuromuscular transmission. Typical clinical features include hypotonia, fatigable weakness, ptosis, and ophthalmoparesis (1, 2). Less frequently, CMS may present with limb girdle weakness (3). Most CMS manifest in the neonatal period or in infancy (4), but adult onset is also possible (5). The pathophysiological mechanisms underlying the different CMS are diverse, including alterations of presynaptic, synaptic or postsynaptic neurotransmission as well as defects of protein glycosylation, endplate development, and maintenance. The majority of CMS result from genetic defects in postsynaptic proteins, with acetylcholine receptor subunits being the most frequently implicated. Presynaptic CMS are much less frequent (6). These are caused by defects in proteins involved in acetylcholine synthesis, its packaging into synaptic vesicles, vesicle docking, and release from the presynaptic nerve terminal (Figure 1). In addition, mutations in genes encoding synaptic space proteins or proteins implicated in neuromuscular synapse development and maintenance can result in presynaptic neuromuscular junction dysfunction (6).

Electrodiagnostic testing is a key component of the assessment of patients with a suspected CMS and can help distinguish CMS from other neuromuscular disorders presenting with similar clinical features. Despite their clinical and molecular heterogeneity, CMS are characterized by a compromised safety margin of neuromuscular transmission. Like acquired disorders of the neuromuscular junction, CMS therefore generally demonstrate a decremental response to low-frequency (2–3 Hz) repetitive nerve stimulation (RNS) and increased jitter or blocking on single-fiber electromyography (SFEMG).

In certain presynaptic disorders of neuromuscular transmission, an increased calcium concentration in the presynaptic nerve terminal can overcome a defect in synaptic vesicle release. Such an increase in calcium concentration may be achieved via high-frequency RNS (20–50 Hz) or maximal voluntary muscle contraction and electrophysiologically results in an increase in the compound muscle action potential (CMAP) amplitude. This phenomenon is referred to as facilitation and is most prominent in the autoimmune Lambert-Eaton myasthenic syndrome (LEMS), where ≥100% increment is observed (7). While facilitation has been traditionally considered the hallmark of presynaptic defects of neuromuscular transmission, this finding is absent in many presynaptic CMS. The presence of facilitation depends on the pathophysiological mechanism compromising acetylcholine quantal release (8). The number of quanta released by a nerve impulse (*m*) represents the product of the number of readily releasable quanta (*n*) and their probability of release (*p*). Thus, various factors altering the number of acetylcholine molecules within a vesicle, the pool of readily releasable quanta, or their probability of release can lead to a presynaptic CMS with or without facilitation (Table 1).

**Table 1:** Responses to RNS in CMS due to mutations in presynaptic proteins and other proteins affecting presynaptic function.

**Disorder**	**Gene**	**Low-frequency RNS**	**High-frequency RNS or MVC**	**References**
Synaptobrevin-1-CMS	*VAMP1*	Decrement Increment (33–60%)	Increment (207–780%)	(9, 10)
Munc13-1-CMS	*UNC13A*	Decrement	Increment (400%)	(11)
Synaptotagmin 2-CMS	*SYT2*	Decrement No decrement	Increment (21–420%)	(12–14)
Laminin α5-CMS	*LAMA5*	Decrement	Increment (250%)	(15)
Agrin-CMS	*AGRN*	Decrement	No increment Increment (23–285%)	(16–22)
Choline acetyltransferase-CMS	*CHAT*	Decrement after conditioning at 10 Hz or maximal contraction Decrement at baseline No decrement	No increment	(23–28)
Vesicular acetylcholine transporter-CMS	*SLC18A3*	Decrement at baseline Decrement after 10 s maximal contraction	ND	(29)
Synaptosomal-associated protein 25-CMS	*SNAP25*	Decrement	ND	(30)
High-affinity choline transporter-CMS	*SLC5A7*	Decrement at baseline Decrement after conditioning at 20 Hz No decrement	ND	(31, 32)
Myosin 9a-CMS	*MYO9A*	Decrement No decrement	ND	(33)
Rabphilin 3A-CMS	*RPH3A*	No decrement	Increment (30%)	(34)
Paucity of synaptic vesicles and reduced quantal release	Unknown	Decrement	No increment	(35)
Laminin β2-CMS	*LAMB2*	Decrement	No increment	(36)

*CMS, congenital myasthenic syndrome; ND, no data; RNS, repetitive nerve stimulation; MVC, maximum voluntary contraction*.

Herein, we focus on the electrophysiological features of presynaptic CMS and their underlying molecular mechanisms. We particularly aim to highlight how the lack of facilitation may still stem form a presynaptic defect and to increase awareness of presynaptic CMS without facilitation. It should be noted that due to the extreme rarity of many of these disorders, it is possible that new cases may emerge with mutations in the same genes, but clinical and electrophysiological phenotypes deviating from previous reports.

## Presynaptic CMS With Facilitation

### Synaptobrevin-1-CMS

Synaptobrevin-1, also called vescicle-associated membrane protein 1 (*VAMP1*), is a component of the soluble N-ethylmaleimide-sensitive factor attachment protein receptor (SNARE) complex. The SNARE complex, which additionally consists of synaptosomal-associated protein 25 (SNAP25) and syntaxin, constitutes the core of the synaptic vesicle docking, priming and fusion machinery (37). *Vamp1*-null mice were found to lack movements due to compromised neuromuscular transmission (38). Homozygous missense or frameshift mutations in *VAMP1* were detected in 3 unrelated CMS families (9, 10). Patients presented in the neonatal period with hypotonia, severe weakness, feeding and respiratory difficulties, delayed motor development and contractures. One patient showed joint hyperlaxity. Affected individuals had low CMAP amplitudes and an incremental response (up to 780%) to 20 Hz RNS. One individual had a decremental response to low-frequency RNS, while two others had incremental responses of 33 and 60%. Myopathic abnormalities on needle examination were also reported in several patients. Parameters of quantal release were not studied in these patients. An incremental response to high-frequency RNS was also identified in *Vamp1-*null mice, supporting the presence of a presynaptic defect of neuromuscular transmission (10). Transfection of mutant synaptobrevin-1 into bovine chromaffin cells significantly decreased depolarization-elicited exocytosis (9). Pyridostigmine was beneficial in most patients.

### Munc 13-1-CMS

Munc13-1, encoded by *UNC13A*, is expressed at the neuromuscular junction, where it facilitates assembly of the SNARE complex (39, 40). Loss of Munc13 in mice prevents docking of synaptic vesicles to active zones (40). Munc13 is also expressed at glutamatergic synapses in the central nervous system (39). A single patient with a CMS due to a homozygous *UNC13A* non-sense mutation has been reported to date (11). This patient had severe hypotonia at birth and required ventilatory support. In addition, she had cortical hyper-excitability, microcephaly, a thin corpus callosum, contractures, and dysmorphism. She died of respiratory failure at age 50 months (11). Baseline CMAP amplitudes were reduced and a decremental response (20–40%) was observed on low-frequency RNS, while 50 Hz RNS produced an incremental response of up to 400%. *In vitro* electrophysiological studies demonstrated that the defect of neuromuscular transmission was attributable to reduced quantal content resulting from the low number of readily releasable quanta, while the probability of quantal release was preserved (11). This finding differentiates Munc13-1-CMS from LEMS, in which there is a reduced probability of quantal release (41). Therapy with pyridostigmine and 3,4-diaminopyridine (3,4-DAP) increased CMAP amplitudes, but only the latter improved the patient's symptoms.

### Synaptotagmin 2-CMS

Synaptotagmin 2 (*SYT2*) is expressed on the synaptic vesicle membrane, where it acts as a calcium sensor, interacting with SNAP25 to promote calcium-triggered synaptic vesicle release (42). The role of synaptotagmin-2 at the neuromuscular junction was demonstrated by the reduced evoked neurotransmitter release observed in synaptotagmin 2-deficient mice (42). To date, heterozygous *SYT2* missense mutations have been reported to cause CMS in 3 families (12–14). Affected patients had pes cavus, hammertoes, and distal lower limb weakness and atrophy since childhood. In addition, some had proximal weakness and extraocular muscle involvement. Deep tendon reflexes were diminished in all patients at baseline but elicitable after exercise in some. Electrophysiological studies revealed reduced CMAP amplitudes, a decremental response to low frequency RNS in all but one patient, and an incremental response of up to 420% following 10 s of maximum voluntary contraction. The duration of the incremental response was prolonged, lasting up to 60 min (12). No *in vitro* electrophysiological studies of neuromuscular transmission are available in SYT2-CMS. A drosophila model revealed that the synaptotagmin 2 mutation exerted a dominant negative effect on synaptic transmission by abolishing calcium-triggered neurotransmitter release (13). It was speculated that the facilitation could result from increased calcium concentrations overcoming the decreased calcium-binding affinity of mutated synaptotagmin. The reason for the prolonged post-tetanic facilitation, longer than observed in LEMS and botulism, remains poorly understood. In the reported patients, pyridostigmine provided no clinical benefit. 3,4-DAP resulted in improved motor function but had no impact on CMAP amplitude or the degree of post-exercise facilitation. SFEMG however showed improvement of jitter and blocking during treatment with pyridostigmine and further improvement during treatment with 3,4-DAP (12).

### Laminin α5-CMS

Laminins are heterotrimeric extracellular matrix proteins composed of combinations of α, β, and γ subunits. They interact with receptors on the sarcolemmal membrane and serve both a structural and signaling role in axon growth and repair (43). Laminin α5 (*LAMA5*) is one of the subunits selectively expressed at the neuromuscular junction (44). Mice deficient in laminin α5 displayed delayed neuromuscular junction maturation (45). One patient has been reported with a homozygous *LAMA5* mutation leading to a CMS associated with myopia and facial tics. A significant decrement was seen on low-frequency RNS, while 30 s of maximum muscle contraction resulted in a 250% increment of CMAP amplitude (15). *In vitro* microelectrode recordings revealed a severe reduction of mean endplate potential (EPP) quantal content, which was attributable to a failure of nerve stimulation to elicit EPPs at a majority of neuromuscular junctions. This defect was partially corrected with 3,4-DAP. Ultrastructural studies showed nerve terminals that were smaller than their respective post-synaptic endplates, entirely absent, or encased by Schwann cell projections, similar to the ultrastructural findings observed in acetylcholinesterase deficiency. There was also a moderate reduction of the density of synaptic vesicles. The partial apposition of the nerve terminal to the post-synaptic endplate was reminiscent of the changes seen in early development in laminin α5-deficient mice. While not strictly a presynaptic CMS, laminin α5 deficiency is thus included here due to the occurrence of post-exercise facilitation in this disorder.

### Agrin-CMS

Agrin (*AGRN*) is an extracellular matrix protein crucial for neuromuscular junction development and maintenance (46). It is secreted by the presynaptic nerve terminal and interacts with the postsynaptic LDL Receptor Related Protein 4 (LRP4) receptor. LRP4 subsequently activates muscle-specific kinase (MuSK), inducing aggregation of acetylcholine receptors at the neuromuscular junction (46). Mutations in *AGRN* have been reported to cause a recessive CMS (16–22). Symptoms manifested in the first or second decade of life. Most patients displayed distal muscle weakness and atrophy, with or without facial weakness, and developed proximal weakness later in the course of the disease (19). Other patients presented with generalized limb weakness (21). All patients showed a decremental response to low-frequency RNS. Post-exercise increment up to 285% was observed in some patients (19) but not in others (17). Intracellular microelectrode studies of neuromuscular transmission were not performed. The mutated agrin exhibited reduced acetylcholine receptor clustering activity and impaired neuromuscular junction maintenance (17, 19, 21). Some patients responded positively to ephedrine and others to salbutamol. Cholinergic drugs were ineffective. Similar to laminin α5, agrin-CMS is included here due to the occurrence of post-exercise facilitation in some patients.

## Presynaptic CMS Without Documented Facilitation

### Choline Acetyltransferase-CMS

Choline acetyltransferase (*CHAT*) is the enzyme catalyzing the synthesis of acetylcholine from choline and acetyl-CoA in the presynaptic nerve terminal. Recessive mutations in *CHAT* cause a CMS with episodic apneas and intermittent exacerbations. This is the most common presynaptic CMS, accounting for 5% of all CMS (6). It was also the first presynaptic CMS characterized at molecular level (23). The apneic spells manifest in infancy or childhood and patients can have minimal or no myasthenic symptoms between spells (24). Different *CHAT* mutations reduce enzyme activity by reduced expression, altered kinetics, or impaired thermal stability. Some *CHAT* mutations have an allosteric effect (25). Patients with CHAT-CMS may have a decrement on low frequency RNS. Between episodic exacerbations however, most patients display no decrement at baseline because the nerve terminal contains stores of acetylcholine. In such cases, SFEMG can unmask the defect of neuromuscular transmission. Conditioning with 10 Hz RNS for 5 min can also trigger a decremental response on low-frequency RNS and a drop in CMAP amplitude. While the latter can also be observed in other CMS, including postsynaptic CMS, it is the subsequent slow post-subtetanic recovery of CMAP amplitude over up to 30 min that suggests a defect in acetylcholine resynthesis (26, 47). CHAT-CMS patients display no facilitation on high-frequency RNS. *In vitro* electrophysiological studies demonstrated miniature endplate potentials (MEPP) of essentially normal amplitude and normal EPP quantal content in rested muscle. Ten Hz RNS for 5 min however reduced the MEPP amplitude by ~50% and resulted in a rapid decrease of EPP amplitude, which returned to baseline over more than 10 min, consistent with insufficient acetylcholine resynthesis (2, 23). In regard to treatment, cholinesterase inhibitors can control crises (27, 28) and some patients may respond to salbutamol or 3,4-DAP as adjunctive therapy, while others do not (27). Monitoring for life-threatening apneas is essential, especially early in life.

### Vesicular Acetylcholine Transporter-CMS

The vesicular acetylcholine transporter, encoded by the *SLC18A3* gene, is responsible for transporting acetylcholine into synaptic vesicles. Of note, *SLC18A3* is located within the first intron of the *CHAT* gene. The crucial role of the vesicular acetylcholine transporter was demonstrated in *Slc18a3* knockout and knockdown mice, which display neuromuscular junction dysfunction and die of respiratory failure or develop weakness responsive to cholinesterase inhibitors (48, 49). Recessive mutations in *SLC18A3* were detected in two unrelated individuals with CMS manifesting with apneic crises, ptosis, opthalmoplegia and fatigable weakness (29). One of the two patients had learning difficulties. Low-frequency RNS resulted in a severe decremental response in one patient, while in the other patient, a decremental response was only seen after 10 s of isometric contraction. No facilitation was described. *In vitro* electrophysiological studies of neuromuscular transmission were not performed. Pyridostigmine partially improved the myasthenic symptoms and addition of 3,4-DAP and ephedrine resulted in further clinical improvement.

### Synaptosomal-Associated Protein 25-CMS

The plasma membrane-associated SNAP25 exists in two different isoforms, designated SNAP25A and SNAP25B, each characterized by distinct patterns of cellular and anatomical localization and developmental expression. The more abundant isoform, SNAP25B, is expressed at both central and peripheral nervous system synapses (50, 51). *In vitro* studies showed that SNAP25 deficiency abolishes vesicle priming and fast calcium-triggered exocytosis (52). A missense mutation in SNAP25B was identified in a patient with a CMS associated with cortical hyperexcitability, ataxia and intellectual disability (30). The patient's symptoms manifested *in utero*; she was born with contractures and subsequently exhibited motor developmental delay. She displayed a decremental response to low-frequency RNS. *In vitro* analysis of neuromuscular transmission showed that the quantal content of EPP and the number of readily releasable quanta had an almost bimodal distribution, with some being reduced while others were normal or increased. In addition, the probability of quantal release was reduced to 63% of normal. MEPP frequency was decreased as well, suggesting that the mutation also affects spontaneous vesicle exocytosis. The *in vitro* electrophysiological finding of a reduced probability of quantal release would predict the presence of facilitation upon tetanic stimulation, but responses to high-frequency RNS or maximum voluntary muscle contraction were not reported. The pathogenic role of the *SNAP25* mutation was demonstrated by the compromised calcium-driven SNARE complex assembly observed in the presence of the mutated SNAP25 and by reduced depolarization-evoked exocytosis in bovine chromaffin cells transfected with the mutant *SNAP25B*. 3,4-DAP improved patient's muscle strength, while pyridostigmine was not beneficial.

### High-Affinity Choline Transporter-CMS

Cholinergic neurotransmission is ended by cleavage of acetylcholine by acetylcholinesterase within the synaptic cleft. Choline is then taken up into the nerve terminal by the sodium-dependent high affinity choline transporter solute carrier family 5 member 7 (*SLC5A7*), also known as choline transporter 1. Choline is used for resynthesis of acetylcholine. The crucial role of the choline transporter in neuromuscular transmission was demonstrated in choline transporter knockout mice, which have breathing abnormalities and die shortly after birth. These mice have loss of spontaneous and evoked responses at the neuromuscular junction as well as developmental abnormalities of the neuromuscular synapse (53). Recessive mutations in *SLC5A7* were found to cause a CMS with episodic apneas in 8 patients from 7 unrelated families (31, 32). Most affected patients presented at birth or in infancy with apneas, ophthalmoparesis, ptosis, bulbar weakness, and subsequent motor developmental delay. Two siblings were more severely affected, presenting antenatally with polyhydramnios and arthrogryposis. The more mildly affected patients with episodic apneas had a favorable prognosis. The phenotype observed in this disorder is similar to that caused by mutations in *CHAT*, which is also involved in acetylcholine resynthesis. Decremental responses to low-frequency RNS were found in 5 of 6 patients, including one in whom a decremental response was only seen after 10 s of conditioning with 20 Hz RNS. No *in vitro* microelectrode studies of the neuromuscular junction were performed. Expression studies demonstrated that the mutations lead to near-total loss of choline transporter activity. In addition to causing a CMS, dominant mutations in *SLC5A7* have been reported to cause distal hereditary motor neuropathy type VII A (DHMN-VII A), which is characterized by vocal cord paresis (54). While RNS in DHMN-VII patients did not reveal a decrement, increased jitter was reported on SFEMG, although this could result from the presence of immature neuromuscular junctions in the setting of reinnervation (55). Meanwhile, no neurogenic changes were described in SLC5A7-CMS patients (31). Most patients with SLC5A7-CMS responded favorably to cholinesterase inhibitors.

### Myosin 9A-CMS

Myosin 9A (*MYO9A*) is an unconventional myosin expressed in the axons of motor neurons and at the neuromuscular junction. Unlike previously-discussed proteins causing presynaptic CMS, myosin 9A is not directly involved in neuromuscular transmission. Unconventional myosins are molecular motors that bind to and move along the actin cytoskeleton, serving functions such as cargo transport, actin organization, and cell motility (56). Three patients have been described with a CMS caused by recessive mutations in *MYO9A*. All patients had prenatal or neonatal onset of symptoms and presented with hypotonia, ptosis, ophthalmoparesis, bulbar weakness, motor developmental delay, and recurrent respiratory crises, often triggered by infections. Within one affected family, several of the proband's siblings had died of respiratory failure within the first year of life. Cognitive impairment was also present in some patients. The presence of an impairment of neuromuscular transmission was demonstrated by a decremental response to low-frequency RNS in one patient, while the other two were found to have increased jitter on SFEMG. *In vitro* experiments found that *MYO9A* knockdown alters neurite extension and branching in cultured neurons, while knockdown in zebrafish leads to aberrant growth of motor neuron axons during neuromuscular junction formation (33). More recent studies in a mouse motor neuron-derived cell line showed that myosin 9A exerts a role in neuronal cytoskeleton maintenance and protein secretion. Myosin 9A mutations were found to result in impaired agrin secretion (57). Pyridostigmine improved symptoms in all 3 patients. In addition, one patient benefited from 3,4-DAP, while 3,4-DAP and fluoxetine precipitated respiratory crises in another.

### Rabphilin 3A-CMS

Rabphilin 3A (*RPH3A*) is a membrane trafficking protein that plays a role in synaptic vesicle docking, re-priming and endocytosis. It has been suggested that rabphilin 3A acts as a regulator of synaptic vesicle release, but its precise functions remain incompletely understood (58, 59). While rabphilin knock-out mice lack any overt phenotype, cultures of hippocampal neurons from these animals demonstrated an accelerated recovery from synaptic depression (60). Compound heterozygous variants in *RPH3A* were recently identified in one patient with a neurological phenotype of childhood-onset fatigable weakness without extraocular muscle involvement as well as learning disabilities, tremor and ataxia (34). The patient did not demonstrate a decremental response to low-frequency RNS but did have increased jitter on SFEMG and a 30% incremental response to 30 Hz RNS. *In vitro* intracellular microelectrode studies of neuromuscular transmission were not performed. Ultrastructural studies of the neuromuscular junction showed a decreased density of synaptic vesicles, which had a variable shape. There was also an increase in non-vesicular membranes and degenerative lamellar bodies in the nerve terminals. Expression studies showed that only one of the detected variants impairs the interaction between rabphilin 3A and 14-3-3, a regulator of synaptic transmission. The patient's response to cholinergic drugs was not described. The role of rabphilin 3A in causing CMS therefore requires further investigation.

### Paucity of Synaptic Vesicles and Reduced Quantal Release

Walls et al. described a 23-year-old patient with a CMS characterized by infantile onset of fatigue, weakness, ptosis, ophthalmoparesis and recurrent episodes of bulbar dysfunction. The patient responded to cholinesterase inhibitors. Electrodiagnostic testing revealed a decremental response to low-frequency RNS, while high-frequency RNS did not result in an incremental response. *In vitro* electrophysiological testing demonstrated a reduced EPP quantal content attributable to a reduction in the number of readily releasable quanta to 20% of normal, with a preserved probability of release (35). Electron microscopy confirmed an 80% reduction in synaptic vesicle density. Although the molecular basis of the syndrome was not identified, the ultrastructural and electrophysiological findings showed a paucity of synaptic vesicles, explaining the lack of facilitation on high frequency RNS. It was suggested that the paucity of synaptic vesicles could result from impairment of synaptic vesicle formation, axonal transport or membrane recycling.

### Laminin β2-CMS

Laminin β2 (*LAMB2*) is another laminin subunit expressed at the neuromuscular junction as well as in the glomerular basement membrane and ocular structures. Biallelic loss of function mutations in *LAMB2* cause Pierson syndrome, which is characterized by neonatal onset of nephrotic syndrome and ocular abnormalities, often along with hypotonia and developmental delay. The classical phenotype is severe, resulting in death in infancy or early childhood (61), which may limit assessment of neuromuscular involvement. Long-term survival however has been reported in some patients, one of whom displayed a CMS phenotype (36). This patient presented in the neonatal period with nephrosis and episodes of respiratory distress. She displayed motor developmental delay, severe proximal weakness, ptosis and ophthalmoparesis. Electrodiagnostic testing revealed a 24% decrement on low-frequency RNS. Unlike LAMA2-CMS, facilitation was absent and high-frequency RNS worsened the decrement. *In vitro* microelectrode recordings demonstrated an 82% reduction of EPP quantal content as well as a reduction of MEPP amplitude and frequency. Ultrastructural examination demonstrated a reduced size of presynaptic nerve terminals, which were encased by Schwann cells. Synaptic clefts were widened and invaded by Schwann cell processes. Treatment with cholinesterase inhibitors resulted in a severe exacerbation of weakness requiring ventilatory support, but ephedrine was beneficial. Although laminin β2 is a synaptic space protein, it is listed here because the reduced quantal content suggests the presence of presynaptic dysfunction.

## Conclusions

Presynaptic CMS can be caused by mutations in a number of genes with different functions, leading to different electrophysiological findings. Of these, CHAT deficiency is the most common and displays a distinctive electrophysiological phenotype, with a decremental response often absent at baseline but uncovered by conditioning with 10 Hz RNS for 5 min, followed by slow recovery of the CMAP amplitude. This underscores the importance of performing provocative testing to unmask the defect of neuromuscular transmission in patients with a suspected CMS. Other presynaptic CMS are caused by mutations in other proteins involved in acetylcholine resynthesis and packing, or in the SNARE complex and related proteins. In these conditions, the presence of facilitation depends on the mechanism responsible for the compromised quantal release. The absence of facilitation is thus not limited to post-synaptic disorders of neuromuscular transmission. A sound understanding of the pathophysiology of these disorders is therefore key in the interpretation of electrodiagnostic findings in CMS patients.

## Author Contributions

SN and MM jointly reviewed the literature and drafted the manuscript.

### Conflict of Interest Statement

MM receives an honorarium as associate editor of Neurology Genetics and an MDA care center grant. The remaining author declares that the research was conducted in the absence of any competing relationships, whether personal, professional, commercial, financial or otherwise, that could be construed as a potential conflict of interest.
